# Characteristics of Kenyan women using HIV PrEP enrolled in a randomized trial on doxycycline postexposure prophylaxis for sexually transmitted infection prevention

**DOI:** 10.1186/s12905-023-02413-0

**Published:** 2023-06-03

**Authors:** Kevin Oware, Lydia Adiema, Bernard Rono, Lauren R. Violette, R. Scott McClelland, Deborah Donnell, Caitlin W. Scoville, Josephine Odoyo, Jared M. Baeten, Elizabeth Bukusi, Jenell Stewart

**Affiliations:** 1 Kenya Medical Research Institute (KEMRI), Kisumu, Kenya; 2 Departments of Global Health, University of Washington, Seattle, United States; 3 Departments of Medicine (Infectious Diseases), Seattle, United States; 4 Departments of Epidemiology, Seattle, United States; 5 Departments of Obstetrics and Gynaecology, Seattle, United States; 6 Fred Hutchinson Cancer Research Center, University of Washington, Seattle, United States; 7 Gilead Sciences, Foster City, United States; 8 Hennepin Healthcare, Minneapolis, MN United States

**Keywords:** STI, Cisgender young women, HIV, PrEP, Doxycycline post-exposure prophylaxis

## Abstract

**Introduction:**

The global incidence of sexually transmitted infections (STIs) has been rapidly increasing over the past decade, with more than one million curable STIs being acquired daily. Young women in sub-Saharan Africa have a high prevalence and incidence of both curable STIs and HIV. The use of doxycycline as a prophylaxis to prevent STIs is promising; however, clinical trials, to date, have only been conducted among men who have sex with men (MSM) in high-income settings. We describe the characteristics of participants enrolled in the first trial to determine the efficacy of doxycycline post-exposure prophylaxis (PEP) to reduce STI incidence among women taking daily, oral HIV pre-exposure prophylaxis (PrEP).

**Methods:**

This is an open-label 1:1 randomized clinical trial on the efficacy of doxycycline PEP compared with standard of care (e.g., quarterly STI screening and treatment) to reduce incident bacterial STIs – *Neisseria gonorrhoeae*, *Chlamydia trachomatis*, and *Treponema pallidum* – among Kenyan women aged ≥18 and ≤30 years. All were also taking HIV pre-exposure prophylaxis (PrEP). We describe the baseline characteristics, STI prevalence, and STI risk perception of participants.

**Results:**

Between February 2020 and November 2021, 449 women were enrolled. The median age was 24 years (IQR 21–27), the majority were never married (66.1%), 370 women (82.4%) reported having a primary sex partner, and 33% had sex with new partners in the three months prior to enrolment. Two-thirds (67.5%, 268 women) did not use condoms, 36.7% reported transactional sex, and 43.2% suspected their male partners of having sex with other women. Slightly less than half (45.9%, 206 women) were recently concerned about being exposed to an STI. The prevalence of STIs was 17.9%, with *C. trachomatis* accounting for the majority of infections. Perceived risk of STIs was not associated with the detection of an STI.

**Conclusion:**

Young cisgender women using HIV PrEP in Kenya and enrolled in a trial of doxycycline postexposure prophylaxis had a high prevalence of curable STIs and represent a target population for an STI prevention intervention.

## Introduction

Global trends reveal a rapid increase in the incidence of sexually transmitted infections (STIs) over the past decade, with more than one million curable STIs acquired daily [1]. In 2020, the World Health Organization (WHO) estimated 374 million new infections of four curable STIs, *Chlamydia trachomatis*, *Neisseria gonorrhoeae*, *Treponema pallidum*, and *Trichomonas vaginalis* [2, 3]. Young women in sub-Saharan Africa face a high prevalence of curable STIs and HIV [4] and limited data from HIV PrEP trials suggests high incidence rates [5]. STIs can severely affect mortality and morbidity for cisgender women by causing conditions such as tubal infertility, chronic pelvic pain, pelvic inflammatory disease, ectopic pregnancy, post-partum endometriosis, adverse neonatal outcomes, and an increase in susceptibility to HIV [6, 7]. Women are more biologically predisposed to complications from STIs than men [8]. Several studies conducted in sub-Saharan Africa reveal higher STI prevalence among younger women compared to their age-matched male peers and older women [8, 9]. In the region, the cultural, economic, and social marginalization of women contributes to the risk of HIV and STIs [10, 11], in part by rendering the negotiation of preventive measures such as condom use, abstinence, and partner notification ineffective [5, 12].

Taking antibiotics following sexual exposure to prevent bacterial STIs places preventive care in the hands of the user. Interventions that are individually controlled are greatly needed, especially for women, and the use of doxycycline as a post-exposure prophylaxis (PEP) has been proposed as a novel STI prevention strategy [13]. Doxycycline is already standardly used as prophylaxis to prevent infections such as malaria, lyme, and leptospirosis [14, 15]. A recent open-label clinical trial of doxycycline PEP among men who have sex with men (MSM) who were using HIV pre-exposure prophylaxis (PrEP) in France found a 47% relative reduction in bacterial STIs overall and a greater reduction specifically for *C. trachomatis* (70%) and *T. pallidum* (73%) [10]. Doxycycline PEP was well-tolerated in that study [18]. Several clinical trials of doxycycline as PEP or PrEP among MSM are ongoing worldwide to test this initial finding.

Studies among young African women have evaluated the association between HIV incidence and perception of HIV risk with disparate results [16–18]. STI risk perception and the prevalence of STIs among women receiving HIV prevention care, which is limited to syndromic management of STIs and daily oral PrEP, have not yet been described. Although women disproportionately bear the burden of adverse sequelae of curable STIs, trials on doxycycline PEP in this population have not yet been completed. The doxycycline postexposure prophylaxis (dPEP) Trial is an open-label, randomized clinical trial evaluating the efficacy of doxycycline PEP for STI prevention (*C. trachomatis*, *N. gonorrhoeae*, and *T. pallidum*) in Kisumu, Kenya, and is the first study to assess the efficacy of doxycycline PEP in cisgender women. In this paper, we describe the baseline characteristics of the dPEP Trial population.

## Methods

### Ethics statement

Before implementation, ethical approval of the protocol was received from Kenya Medical Research Institute’s Scientific Ethics Review Unit (KEMRI-SERU) and the University of Washington’s Institutional Review Board. Written informed consent to participate in the study was obtained from all participants and legal guardians of the illiterate participants before enrolment. All methods were carried out in accordance with the relevant guidelines and regulations. The trial was registered on 08/08/2019 at ClinicalTrials.gov, number NCT04050540[20].

### Study design

This is an open-label 1:1 randomized clinical trial evaluating the efficacy of doxycycline PEP to reduce incident curable, bacterial STIs – *N. gonorrhoeae*, *C. trachomatis*, and *T. pallidum* among Kenyan cisgender young women. Inclusion criteria included willingness and ability to give written informed consent, ≥18 and ≤30 years, female sex assigned at birth, HIV seronegative, and a current prescription for HIV PrEP according to the national guidelines of Kenya. Exclusion criteria included pregnancy, breastfeeding, allergy to tetracyclines, on current medications that may impact doxycycline metabolism or that are contraindicated with doxycycline as per the prescribing information, or recent use of prolonged antibiotics (more than a 14-day course) in the month before enrolment, or active clinically significant medical or psychiatric conditions that would interfere with study participation per the discretion of the study investigator. Use of contraception was not required, and those who became pregnant after enrolment were not disenrolled, but among those assigned to the dPEP group, doxycycline was discontinued and only resumed if no longer pregnant or breastfeeding. Participants were primarily recruited from clinics providing HIV PrEP within Kisumu County. Quarterly follow-up visits were scheduled for each participant for 12 months. The study site is situated within a clinic at the Lumumba sub-County hospital in Kisumu, Kenya.

Demographic and behavioural data were electronically collected (REDCap), including questionnaires on STI and HIV risk perception and potential exposures. Biological sample collection (including serum, endocervical and vaginal swabs) and testing were done by trained study clinicians and laboratory technologists on site. Rapid HIV testing was completed using HIV 4th generation combination test (Abbott Determine) followed by confirmation for any positive results using repeat HIV antibody rapid testing (First Response) and then enzyme linked immunosorbent assay (ELISA) testing per Kenyan National Guidelines. Testing for *C. trachomatis* and *N. gonorrhoeae* was done using nucleic acid amplification test (Cepheid GeneXpert or Aptima Combo 2). *T. pallidum* screening was completed using rapid plasma regain (RPR) test (BD Macro Vue) followed by *Treponema pallidum* haemagglutinin (TPHA) assay (Fortress Diagnostics Limited) to confirm positive RPR results [20].

This study is an open-label, randomized clinical trial of doxycycline hyclate (200 mg taken up to 72 hours after sex, with no more than 200 mg each day) to reduce bacterial STIs. Participants were randomized 1:1 to doxycycline PEP vs. standard of care using computer-based randomization (Randomize.net). The trial’s plan to enrol 446 participants was determined based on an anticipated 66 women with new STIs (*N. gonorrhoeae, C. trachomatis*, or early syphilis) occurring in the 12 months of follow-up, corresponding to an annual incidence of 22% in the standard of care arm. The trial was designed to achieve 80% power to detect a 50% reduction in infections in the doxycycline arm compared to standard of care. Full details of the trial protocol can be found in the Supplementary Appendix, available with the full text of this article at https://trialsjournal.biomedcentral.com [21]. All procedures were performed in accordance with relevant guidelines.

We present descriptive statistics, and compared the detection of an STI (*N. gonorrhoeae, C. trachomatis*, and/or *T. pallidum*) at baseline between women, who perceived themselves as being at risk of acquiring an STI in the next three months, and women, who did not self-perceive STI risk. Relative risks and 95% confidence intervals were estimated using relative risk regression via modified Poisson regression with robust standard errors. All analyses were completed using SAS version 9.4 (SAS Institute, Cary, NC, USA).

## Results

**Fig. 1:**
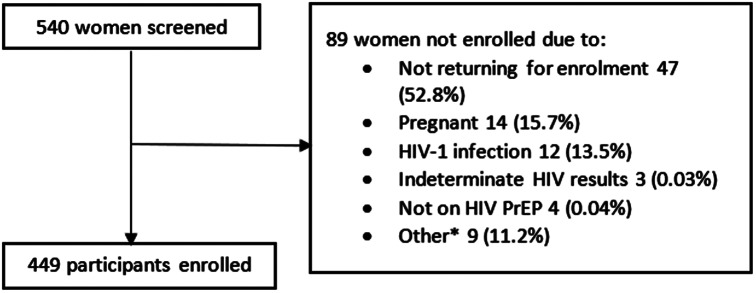
Flow diagram of participant screening and enrolment. (*Other includes age out of study range, breastfeeding, declined study procedures, comorbidities, and not sexually active)

Between February 2020 and November 2021, 540 cisgender women were screened for study eligibility and 449 were enrolled (Fig. 1). The screening to enrolment ratio was 1.2:1. The median age of participants was 24 years (IQR 21–27) (Table 1). Most were never married (66.1%, 297 women). One hundred and thirty-eight women (30.7%) had not given birth at baseline. Over three quarters (76.8%, 345 women) had attended at least secondary schooling or higher, and 62.4% (280 women) reported that they earned their own income.

The majority of women enrolled (82.4%, 370 women) reported having a primary sex partner in the three months prior to enrolment, and one-third (33.2%, 149 women) reported sex with a new partner in the past three months. A total of 268 (67.5%) women reported not using a condom in the most recent vaginal sex act. Over half (56.6%, 254 women) had tested for HIV together with their male partners. Recent transactional sex, sex in exchange for goods, gifts, or money, was reported by 36.7% (165 women), and 16.7% (75 women) reported drinking alcohol before sex in the past month.

**Table 1:** Baseline demographic data, sexual behaviour, contraceptive use, and STI prevalence

Variable characteristic	N (%)
**Median age (IQR) years**	24 (21–27)
**Marital status**	
Never married	297 (66.1%)
Married (monogamous)	82 (18.3%)
Married (polygamous)	10 (2.2%)
Separated	52 (11.6%)
Divorced	6 (1.3%)
Widowed	2 (0.4%)
**Has a primary sex partner**	362 (80.6%)
**Had a primary sex partner in 3 months before enrolment**	370 (82.4%)
Tested for HIV together with primary partner	254/370 (68.6%)
**Reported sex with new partners 3 months before enrolment**	149 (33.2%)
**Parity**	
None	137 (30.5%)
1 live birth	172 (38.3%)
2 live births	106 (23.6%)
3 or more live births	34 (7.6%)
**Highest level of education**	
No schooling	1 (0.2%)
Primary school, some or complete	103 (22.9%)
Secondary school, not complete	116 (25.8%)
Secondary school, complete	147 (32.7%)
Attended post-secondary school	82 (18.3%)
**Participant earns own income**	280 (62.4%)
**Transactional sex (prior 3 months)**	165 (36.7%)
**Condom use in most recent vaginal sex act**	129/397* (32.1%)
**Contraceptive type**	
Implant	149 (33.2%)
None	100 (22.3%)
DMPA	98 (21.8%)
Condoms	65 (14.5%)
Oral contraceptives	20 (4.5%)
Intrauterine Device	11 (2.4%)
Emergency contraceptives & Condoms	6 (1.3%)

*Fifty-two participants did not have vaginal sex in the 3 months prior to enrolment

More than half of the women were using long-acting reversible contraceptives: implant 149 (33.2%), injectable depot-medroxyprogesterone acetate 98 (21.8%), and intrauterine device 11 (2.4%). One hundred women (22.3%) reported not using any modern contraceptive method. Symptoms commonly associated with doxycycline were reported by some participants prior to randomization, including nausea 38 (8.5%), diarrhoea 19 (4.2%), vomiting 13 (2.9%), and photosensitivity 4 (0.9%).

Overall, 17.9% of women (80/448) had any bacterial STI, including 14.1% (63/448) with *C. trachomatis*, 5.8% (17/448) with *N*. *gonorrhoeae*, and 0.4% (2/449) with *T. pallidum*, where two participants (0.4%) presented with both *N. gonorrhoeae* and *C. trachomatis* (Table 2).

**Table 2:** Baseline STI prevalence among cisgender women enrolled in a trial of doxycycline postexposure prophylaxis in Kisumu, Kenya

STI type	n/N(%)
*C. trachomatis*	63 /448** (14.1%)
*N. gonorrhoeae*	17 /448** (3.8%)
*T. pallidum*	2 /449 (0.4%)
STI co-infection	2/448 (0.4%)
Any STI	80 /448** (17.9%)

**One participant enrolled without baseline vaginal swab collection

**Fig. 2:**
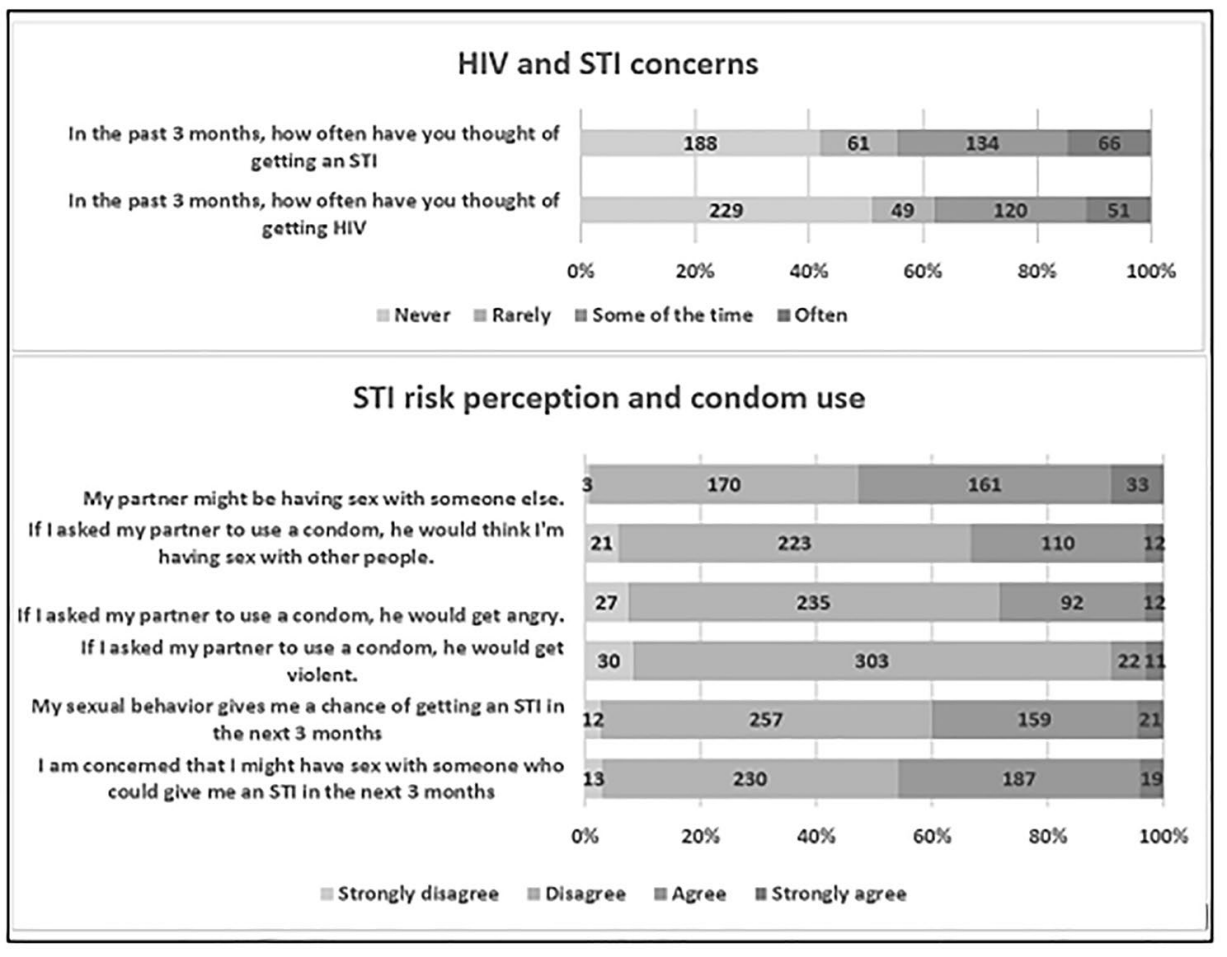
Risk concerns and perceptions among the 449 women taking HIV PrEP and enrolled in the dPEP Trial

Participants reported being often or sometimes concerned with contracting STIs (44.5%, 200 women) and HIV (38.1%, 171 women) (Fig. 2). Cumulatively, about half of the participants (45.9%, 206 women) either agreed or strongly agreed that they were concerned they might engage in sex with someone who could infect them with an STI in the next three months. One hundred and eighty women (40.1%) perceived that their sexual behaviour could give them a chance of getting an STI in the next three months. About half (52.9%, 194 women) suspected that their male partners might be having sex with someone else. However, there was no association between women’s self-perceived STI risk and STI detection at enrolment, RR 0.90, 95% (CI 0.60–1.36, P = 0.622).

## Discussion

The trial enrolled 449 young women, who were taking HIV PrEP, and half (224) were randomly assigned to take doxycycline PEP. The use of doxycycline as PEP or PrEP for STI prophylaxis is concurrently being evaluated in multiple ongoing trials in the US and Australia among MSM [22]. The potential for long-term complications that result from a bacterial STI, however, is much greater in cisgender women[7, 11, 23]. The dPEP Trial is among the first to focus on the use of doxycycline prophylaxis for the primary prevention of bacterial STI among women.

Social structures and sexual behaviour that necessitate HIV PrEP use often correlate with a high risk of being exposed to a bacterial STI [4, 5]. These baseline data indicate that women who are taking HIV PrEP are at significant risk of contracting curable STIs with an overall prevalence of 17.9% (80), the majority (63) due to *C. trachomatis*. These data support findings from previous studies which equally indicated a high prevalence of *C. trachomatis* among women of reproductive age in sub-Saharan Africa [9, 18]. Also consistent with other studies in Kenya, *T. palladium* had the lowest prevalence, with only two participants testing positive [24]. These baseline data further suggest the need for STI prevention and treatment integrated into PrEP care in sub-Saharan Africa. Furthermore, among the 540 women, primarily recruited from PrEP care, a significant proportion tested positive for HIV at screening (2.2%, 12 women) highlighting the persistent risk of HIV infection and challenges with adherence to HIV PrEP.

A total of 180 (40.1%) of the cohort identified their sexual behaviours as possibly increasing the risk of STIs with more reporting concerns with the behaviours of their male partners (45.9%, 206 women). Overall, slightly less than half of the women, 200 (44.5%) were concerned about contracting STIs in general. This cohort reported low rates of condom use and fear of conflict with negotiated condom use, highlighting the need for structural interventions beyond individual behaviour to reduce the risk of STIs among women [25]. Moreover, the low rates of condom use and high rates of STIs in this cohort indicate that women need a strategy to prevent STIs that they can control by themselves since condoms are typically controlled by their male partners.

Health complications that may stem from STIs among cisgender women include tubal infertility, chronic pelvic pain, pelvic inflammatory disease, ectopic pregnancy, post-partum endometriosis, adverse neonatal outcomes like premature death and premature delivery, and increased risk of HIV acquisition[6, 7, 23, 26]. Despite the high frequency of sexual activity, not all study participants reported prior live births, and of the 137 women (30.5%) without prior delivery, 100 (72.5%) were not using hormonal contraception at enrolment. Multiple factors could explain this observation and may be due to intentional choice for delayed fertility, frequent use of emergency contraception, induced abortions, miscarriage, and/or infertility due to prior STI. Additionally, a significant number of study participants were using contraceptives, and this indicates that women seeking family planning services may benefit from integrating STI prevention, PrEP care, and family planning.

Women of reproductive age in middle and low-income countries are at increased risk of STI-related complications due to limited access to effective prevention strategies, diagnostic testing, or timely treatment [19, 20]. The need for primary prevention of STIs is of global importance with the highest potential for impact among women in low-resource settings. Overall, young women taking HIV PrEP are at risk of curable STIs and have a demonstrated need for women-centered STI prevention programs.

## Conclusion

Young women using HIV PrEP in Kenya have a high prevalence of bacterial STIs. Should doxycycline PEP be proven to be efficacious at preventing STIs, there is substantial potential for benefit, especially in high-prevalence settings such as among women taking HIV PrEP.

## Data Availability

The datasets generated and/or analysed during the current study are not publicly available due to the fact that the study is still ongoing but will be available once study comes to an end in December 2022 on reasonable request from the corresponding author.
